# Quantitative Assessment of Strabismus Using Cloud AI Computing: Validation Study

**DOI:** 10.2196/79280

**Published:** 2025-11-04

**Authors:** Junxian He, Jiawei Zhang, Zheng Wang, Shrinivas Pundlik, Rui Liu, Gang Luo

**Affiliations:** 1 Schepens Eye Research Institute of Mass Eye & Ear Harvard Medical School Boston, MA United States; 2 Chongqing University Chongqing China; 3 Department of Ophthalmology Eye & ENT Hospital of Fudan University Shanghai China; 4 School of Medicine Jiaxing University Jiaxing China; 5 Key Laboratory of Myopia and Related Eye Diseases Fudan University Shanghai China; 6 Shanghai Key Laboratory of Visual Impairment and Restoration Shanghai China

**Keywords:** strabismus, cloud computing, AI models, artificial intelligence, smartphone, web app

## Abstract

**Background:**

Strabismus measurement is essential in vision assessment and screening. It typically requires skilled clinicians or specialized equipment. Photographic strabismus measurement methods have value in terms of accessibility and convenience of use.

**Objective:**

This study aimed to evaluate Eyeturn Cloud, a cloud-based artificial intelligence (AI) system for measuring strabismus angles based on eye images captured with smartphone cameras under cover test conditions.

**Methods:**

The Eyeturn Cloud web app uses AI models to recognize eyes, eye lid, and iris, and then to segment iris precisely. It then computes strabismus based on ellipse fitting of the iris boundary and corneal reflection. The system was evaluated in patients (without glasses) with manifest strabismus and control participants. Clinicians measured eye deviations using the prism alternate cover test and also captured pictures of their eyes under alternate cover and unilateral cover conditions. The pictures were processed by Eyeturn Cloud.

**Results:**

In total, 79 (mean age 11.9, SD 6.3 years; esotropia: n=15, exotropia: n=55, orthotropia: n=9) participants were enrolled; of which, data were available for 71 participants (8/79, 10.1% processing failure). The range of prism alternate cover test strabismus magnitude was from 78 base in to 78 base out prism diopters (PDs). A strong correlation was found between Eyeturn Cloud and clinical measurements (*R*^2^=0.95; slope=0.91; *P*<.001). Bland-Altman analysis revealed that 95% limits of agreement between the 2 measurements were –20.2 to 14.6 PD. A repeatability test with 15 participants (4 photos each) found a 1.53 PD SD.

**Conclusions:**

The cloud AI web app can compute strabismus angles reliably under alternate and unilateral cover conditions in clinical settings, and its potential for use in telehealth settings needs further evaluation.

## Introduction

Strabismus, a condition where the eyes are misaligned, can cause a wide range of visual dysfunctions in children [1,2] and adults [3]. According to the survey [4], strabismus not only negatively impacts activities of daily living but also causes self-concerns regarding appearance to others, even in most patients without diplopia. Current clinical assessments of strabismus are based on some form of cover testing with prisms to determine the presence and the magnitude of eye deviation. This procedure requires skilled and experienced clinicians using instruments such as a prism bar or synoptophore and also a high degree of cooperation from the patients for accurate assessments.

With the development of digital health care technology, various approaches have been developed for automating or simplifying the strabismus measurement process, including eye tracker–based systems [5-7], virtual reality–based systems [8], and smart glasses [9]. For vision screening purposes [10], some commercial screening devices such as Volk Eye Check, Spot Vision Screener, Plusoptix, iScreen, 2WIN, GazeLab [11], neos [12], and blinq are available. These devices are typically built on optoelectronic technologies and are relatively easier to use than conventional prisms. Some, for example, the blinq device, have shown very high sensitivity and specificity for strabismus detection [13]. However, these devices typically only raise red flags when concerning strabismus is detected and generally do not provide quantitative measures (with some exceptions, eg, 2WIN).

For the reasons of costs, accessibility, and sometimes operating difficulties, dedicated screening devices are not available in many settings, such as resource-limited regions, nursing homes, and in telehealth scenarios [14,15]. In these settings, photographic methods, which just need a picture of patients’ eyes, may be preferable.

Approaches to measure strabismus based on a photograph include using dedicated cameras [16-18], or more recently, using a smartphone camera and flash to take a picture of the eyes and detect strabismus [19-24]. Other automated systems have used different technologies, such as virtual reality headsets or dedicated infrared eye trackers [25,26]. Strabismus assessment based on captured photos using web tools [27] and artificial intelligence (AI) models [28] has been developed. Photographic assessment of strabismus, via mobile apps, cloud computing, and AI algorithms, offers the advantages of accessibility and convenience.

Most photoscreening devices, including smartphone apps, are limited to measuring manifested strabismus, and intermittent patients with strabismus are likely to be missed. The ability to elicit and capture the eye movement based on covering 1 or both eye alternatingly remains a challenging problem for mobile apps and many other photoscreening devices. In fact, in our previous study of the Eyeturn app [19], a stand-alone photographic strabismus measurement tool, we indicated that the cover or uncover feature within the app required offline processing of the videos. In addition to the inability to effectively measure strabismus under cover or uncover or alternating cover (AC) mode, limitations of the prior stand-alone Eyeturn app include on‑device model constraints (only lightweight models capable of running on mobile hardware could be used), uncorrected corneal‑reflex or iris detection errors and lack of quality control, device variability that caused software mismatch with hardware, and inability to reprocess previously saved images (the app only worked with live camera capture).

To overcome this challenge, we have developed a smartphone app (cover test app) to enable automatic picture capture under various cover or uncover conditions [29] and an Eyeturn Cloud web app for strabismus angle calculation based on the uploaded pictures of the eyes. In this study, the cover test app is merely a facilitator for the precise capture of eye movements at the instant of uncovering (see demonstration video in Multimedia Appendix 1). The present cloud system addresses these gaps by using a different workflow for image capture in different cover test modes, which improves processing via various large computer vision models. The current cloud app can be used for retrospective reprocessing of archived images for recalculation and audit.

By separating photo capturing and image analysis, the Eyeturn Cloud web app was developed with the ultimate goal of improving accessibility and reducing dependence on specialized equipment, dedicated apps, or skilled operators for strabismus assessment. While the cover test app may be convenient, it is not strictly necessary to capture images for uploading to Eyeturn Cloud. One can use regular digital cameras or native phone camera apps to take photos, if the goal is to assess manifested strabismus (cover is not needed). Used together with either regular phone camera app or our dedicated cover test app [29], the proposed solution could be potentially used for vision screenings in resource-limited areas and for telehealth settings (such as virtual visits for prescreening or follow-up [14] and for vision rehabilitation [15]), where pictures taken by nonprofessional personnel could be uploaded for quantitative analysis and then reviewed by an ophthalmic expert at remote sites.

Together with other complementary apps for measuring various visual functions (eg, visual acuity and refractive error), Eyeturn Cloud may potentially help in enabling a more accessible and scalable solution in the management of strabismus. For patient subgroups such as children, a rapid, photographic-based method may improve cooperation compared to conventional examinations that require prolonged fixation [30]. Furthermore, obtaining objective, quantitative measurements is critical for monitoring disease progression or treatment response over time, which is a cornerstone of pediatric strabismus management [31]. Access to pediatric ophthalmology is uneven, with notable rural and underserved gaps. Contemporary screening recommendations endorse early detection but often lack scalable, quantitative tools deployable outside specialty clinics. By enabling photographic acquisition with cloud‑based, standardized quantification, our system can provide actionable, reproducible measurements for triage and follow‑up by nonspecialist personnel (eg, school nurses and primary care staff) and in community settings, potentially shortening time‑to‑referral and improving longitudinal care. Importantly, no specialized equipment or specialist presence is required. A simple photograph suffices for measurement and can be acquired in routine environments without dedicated clinical instrumentation.

This study quantitatively evaluates the strabismus measurement approach using the cover test app for image capture and the AI-powered cloud computing paradigm for strabismus angle calculation. The primary objective was to evaluate the cloud-AI platform’s effectiveness in accurately measuring strabismus angles. We hypothesize that the Eyeturn Cloud platform can provide quantitative measurements of strabismus that are highly consistent with the clinical gold standard of cover testing with prism. The primary outcomes for this validation are the coefficient of determination (*R*^2^) from linear regression and the 95% limits of agreement from Bland-Altman analysis. A secondary outcome is the test-retest reliability of the platform’s measurements.

## Methods

### Pipeline of Strabismus Calculation

**Figure 1:**
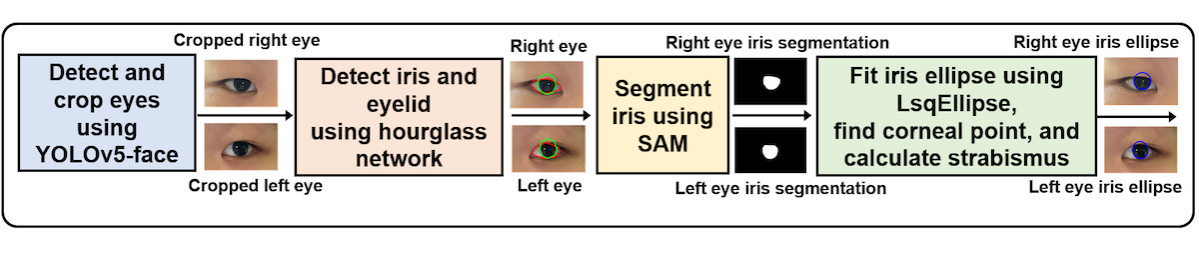
Image processing pipeline of Eyeturn Cloud web app for eye deviation calculation. The processing includes 4 steps—eye localization, iris localization, iris segmentation, iris boundary fitting, and deviation calculation. SAM: Segment Anything Model; YOLO: You Look Only Once.

The entire image processing is executed in a cloud app instance running in Amazon Web Services (AWS). As illustrated in Figure 1, the app includes 4 key steps to calculate eye deviation. First, using the YOLOv5-face (You Look Only Once) model [32], the eyes are detected, localized, and cropped. Next, using an hourglass network [33] trained with a synthesized eye image set [34], the iris is localized in cropped eye images. Using the center of the iris as a reference point, the Segment Anything Model (SAM) [35] is used to segment the visible iris region. Finally, an ellipse is fitted to the segmented iris. The center of the ellipse is considered as the precise location of the iris center. Combining with corneal reflection points, which are detected by simple thresholding, eye deviation can be calculated according to the Hirschberg method [16,20,36]. We did not train YOLOv5-face and SAM models. Pretrained versions were used with default parameters for inference.

### Iris Detection

Iris is detected using a coarse-to-fine approach. After obtaining the coordinates of the eyes, the left and right eye regions are cropped from the input image and resized to 72×120 pixels. The subimages are processed by the hourglass network for localizing the iris and eyelids. The network is a stacked-hourglass network trained on a synthetic eye image set [34]. Detected iris and eyelid landmarks are represented by polygons, which approximate the curves of those eye features. Based on our evaluation, the center of the iris polygon is not precise enough for strabismus calculation. Therefore, the iris center is then selected as the reference point for more precise segmentation using SAM [35], which segments the visible iris region pixel-wise. SAM generates 3 segmentation results ranked by probability. The result with the highest probability is not necessarily the best segmentation. Sometimes it corresponds to the pupil rather than the iris. To address this, we calculate the number of pixels within the area delineated by the iris segmentation results and exclude the pixels that are outside the eyelid polygon. The number of remaining pixels in each segmentation and shape are verified for reasonability. The closest match to the expected iris is chosen as the final segmentation.

Since the iris is partially occluded by eyelids in most cases, the center of the segmented iris is not the true iris center. Therefore, an ellipse is fitted to the boundary of the segmented iris. To achieve a better elliptical fit, we applied morphological opening to the segmented iris to smooth the boundary without significantly altering the area. Then, the segmentation edge of the iris was extracted, retaining only the curved lines on the left and right sides. Using the *LsqEllipse* package [37,38], an ellipse was fitted to these curved lines to represent the shape of the iris, and the center of the ellipse was regarded as the precise center of the iris.

The AI models for ocular landmark detection or segmentation were trained on diverse datasets from different image sources. Therefore, it is anticipated that the strabismus measurement method is hardware agnostic, and no retraining is needed for different acquisition hardware.

### Cloud App: Eyeturn Cloud

**Figure 2:**
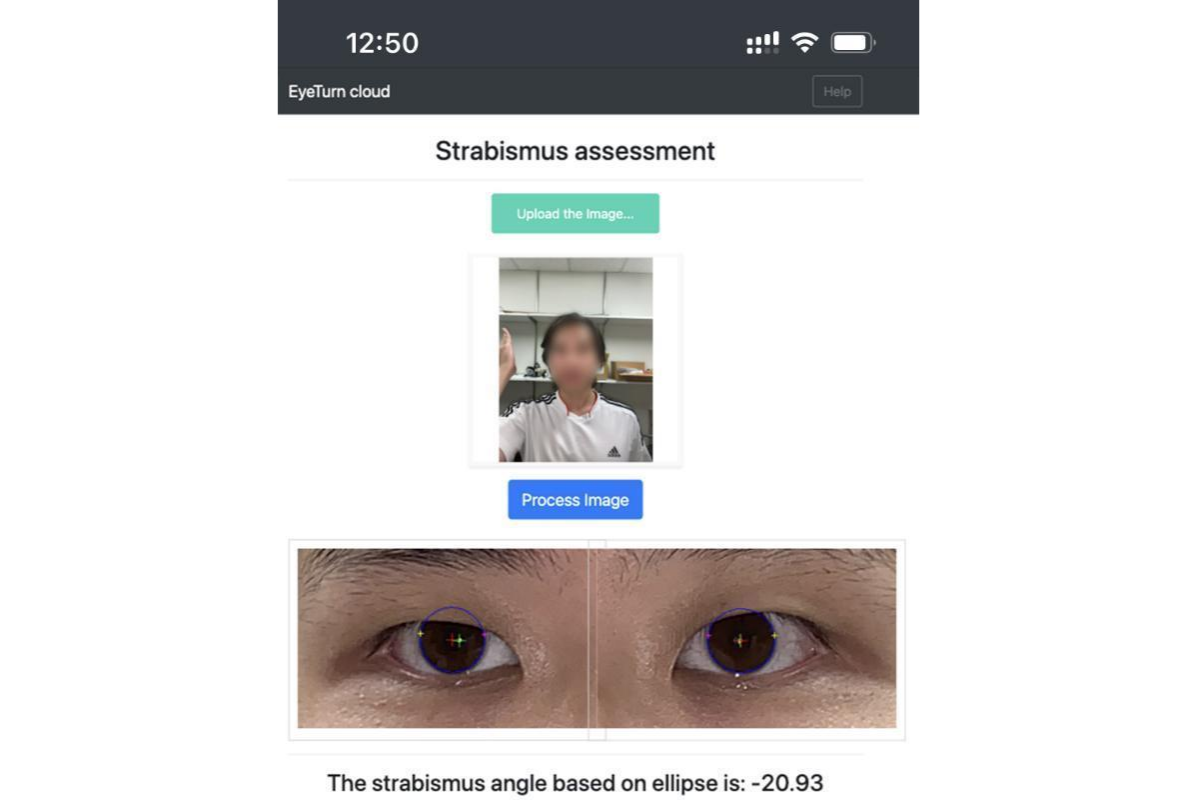
A screenshot of the Eyeturn Cloud interface when the web app was accessed from a phone. The uploaded picture shows that the participant covered his right eye with his hand unilaterally and then quickly uncovered. The ellipse fitting of iris is shown as a blue oval. If the fitting is obviously wrong, users should reject the result.

The front-end of Eyeturn Cloud was developed using Cascading Style Sheets and JavaScript and was initiated with the Python *Flask* package. The Eyeturn Cloud website prompts users to upload a photo, and a “Process image” button appears once the upload is successful. Upon clicking the button, the back-end is activated to perform strabismus calculations (Figure 2). The back-end, developed in Python, was responsible for calculating the strabismus result based on the photo sent from the front-end. Once the calculation is completed, the cropped and processed eye images, along with the strabismus result, are displayed. The user could then verify the accuracy of the strabismus result by reviewing the processed eye images, with the estimated iris ellipse and corneal light reflex points overlaid. The Eyeturn Cloud website is deployed on AWS. We selected a t2.2xlarge instance with 8 virtual central processing units to accelerate strabismus calculation and 32 GB RAM to accommodate the YOLOv5-face, High-Resolution Network, and SAM models. This instance was configured to run on Ubuntu 22.04 as a virtual server to ensure that the Eyeturn Cloud platform works continuously.

### Cover Test App

A specialized app for capturing pictures during cover or uncover or AC tests was developed because the intermittent deviation needs to be computed at the precise moment, just after the cover is removed. Otherwise, there is a risk that the measured deviation via the app is different from the true deviation. The cover test app is based on the idea of detecting the frame when both eyes are simultaneously present [29]. The detection of eyes is done using custom-trained YOLOv5 deep learning model, capable of working in real time on contemporary iPhones. To develop the real-time eye detection model used in our data capture app (the cover test app), we created a custom dataset by collecting face images from public web sources and from our own captures. A total of 1013 eyes were manually annotated from these images. The dataset was split into training (810 eyes), validation (101 eyes), and testing (102 eyes) sets. We trained the model by fine-tuning the official YOLOv5 pretrained checkpoint on our custom dataset, using the source code with its default training parameters.

The procedure to perform cover or uncover or AC tests with the cover test app is similar to the conventional way. The examiner places an occluder in front of one eye, while the other eye fixates on the flash. The occluder is then removed away from the face by the examiner at an appropriate time, revealing both eyes. Once the app detects the presence of both eyes, it saves that frame, which can then be uploaded to Eyeturn Cloud for strabismus angle calculation. Since the app works in real time, there is very little delay in capturing the just-uncovered eye (approximately 33-66 milliseconds). In case of strabismus, the just uncovered eye is likely to be deviated, and this can be analyzed from the corneal reflection in the captured frame. Thus, the app facilitates measurement of intermittent strabismus, but it should be noted that Eyeturn Cloud does not have to be paired with this cover test app. It can process pictures captured through other ways; for instance, a frame extracted from a video.

### Ethical Considerations

The clinical evaluation study was conducted in accordance with the tenets of the Declaration of Helsinki. The study was approved by the institutional review board of the Eye and ENT Hospital of Fudan University (Shanghai, China; approval: 2024126). Written informed consent was obtained from all participants prior to enrollment, including details about the study’s purpose, procedures, potential risks and benefits, and the right to withdraw at any time without repercussions; for children, parental or guardian consent was obtained. Participant selection was not influenced by sex or age. Images were processed locally on a computer (localhost) rather than uploaded to external cloud services like AWS during the study, minimizing data exposure. No identifiable information was shared beyond the research team. Participants did not receive any compensation for their participation. See Multimedia Appendix 2 for a completed checklist related to adherence to accepted guidelines for transparency, reproducibility, and methodological rigor in AI diagnostic studies. Data are not shared as per the conditions placed by the institutional review board to protect patient privacy.

### Sample Size Calculation

From our previous study of the Eyeturn smartphone app [17], the SD of the difference between the app and clinical measurements was 4.1 prism diopters (PDs). Our preliminary analysis showed that our computation system can provide measurements with a reliability of ±3 PDs. Assuming twice the variability in this study and a type I error rate of 0.05 and 80% power, we arrive at a sample size of 64. Considering dropouts and capture errors, the calculated sample size was inflated further by 25% to arrive at a figure of 80 participants as the recruitment target. In total, 79 participants (mean age 11.9, SD 6.3; range 4-42 years) were enrolled from Eye and ENT Hospital of Fudan University (Shanghai, China).

### Participant Recruitment

Patients with strabismus were recruited from the patient cohort treated by the author (RL) at their affiliated ophthalmology clinic. Inclusion criteria were a prior diagnosis of horizontal strabismus (constant or intermittent exotropia or esotropia) and no other visual impairments. Participants were eligible only if the best‑corrected visual acuity (BCVA) in the worse eye was 20/40 or better (Snellen; 0.30 logMAR), using habitual correction or trial‑frame refraction. Strabismic amblyopia was not an automatic exclusion if the BCVA threshold was met. In our cohort, only 3 participants had strabismic amblyopia, and they satisfied the BCVA better than or equal to 20/40 criterion. Therefore, they were included. As our current system is not sufficiently robust yet to process images of eye wearing glasses, patients with refractive errors higher than 1 diopter were excluded, concerning that they may not be able to fixate at the target properly without vision correction during the cover test. Cases of incomitant strabismus, such as paralytic strabismus, or those with a clinically significant vertical deviation (>5 PD) were also excluded. In addition, 9 participants without manifest strabismus were also enrolled to serve as control participants.

### Study Procedures

Clinicians specialized in strabismus measurement performed the prism alternate cover test for near fixation (about 40 cm). In addition, they also took pictures under 3 cover conditions, AC, left eye cover-uncover (LC), and right eye cover-uncover (RC), using the cover test smartphone app as described earlier [29]. Those pictures were uploaded to the Eyeturn Cloud app, and the results are compared with prism alternate cover test measurements.

The image resolution used for this study was 3024 by 4032. The images were captured with a consumer iPhone 11 and 13, with autofocus at a distance of approximately 40 cm from the face.

We prioritized using the strabismus angle calculated from the AC images as the primary result in this study. If the AC image processing of a patient could not yield a result due to missing corneal reflection, SAM segmentation errors, or other issues, we selected the larger absolute value of the strabismus results from the LC or RC images as the final result.

### Outcome Measures

The clinician’s measurement was used as the ground truth, and the cloud computing results were analyzed by linear regression. A Bland-Altman analysis was also conducted to compare the physician’s assessment and the Eyeturn Cloud results.

## Results

**Figure 3:**
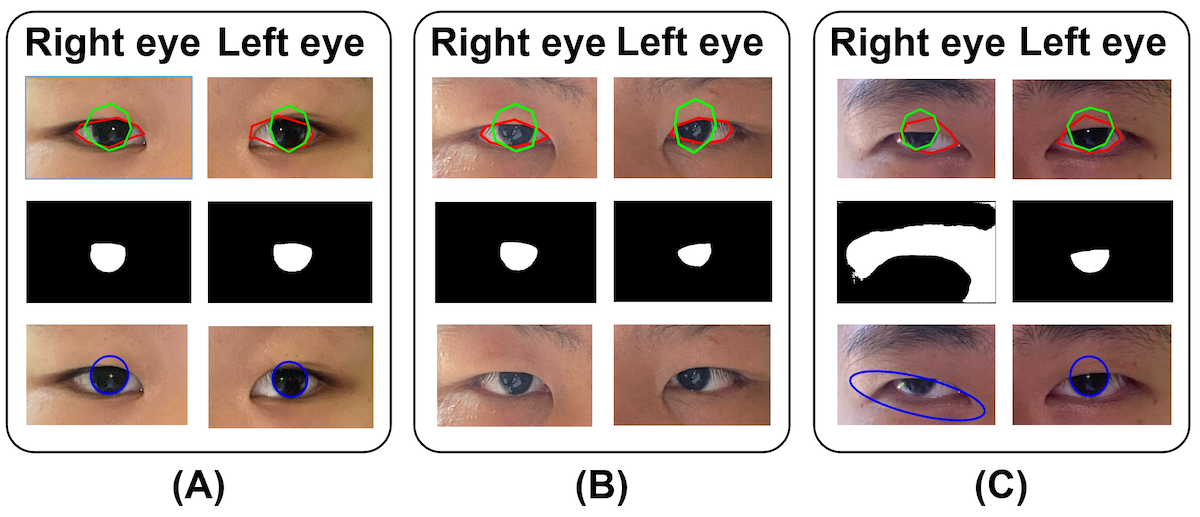
Correct and incorrect examples of Eyeturn cloud processing. Upper panel: coarse iris and eyelid detection, middle panel: fine segmentation of iris, and lower panel: ellipse fitting of iris boundary. (A) A successful processing, (B) corneal reflection could not be detected because of many other reflections in the background, and (C) the segmentation and ellipse fitting of right eye iris are obviously incorrect; therefore, the calculation was rejected.

Among the 79 enrolled participants (including 15 esotropia, 55 exotropia, and 9 orthotropia), strabismus was successfully calculated by the Eyeturn Cloud app for 71 participants. The processing failed for the remaining 8 (10.1%) participants due to failure to detect the corneal reflection for 1 participant and incorrect iris segmentation for 7 participants (Figure 3). The average age of the participants where processing failed was 11 (SD 5) years, which is similar to the age distribution of the entire sample 11.9 (SD 3.6) years. There were 4 male and 4 female participants, and an equal number of base in (43, 32, 77, and 40 PD) and base out (32, 77, 32, and 43 PD) deviations with processing failure. Since the study was conducted in China, the eye color was mostly uniform (deep brown). The failures did not seem to be related to demographic or clinical factors. This type of incorrect detection or segmentation was determined by visually examining the iris ellipse fitting overlaid on processed pictures.

**Figure 4:**
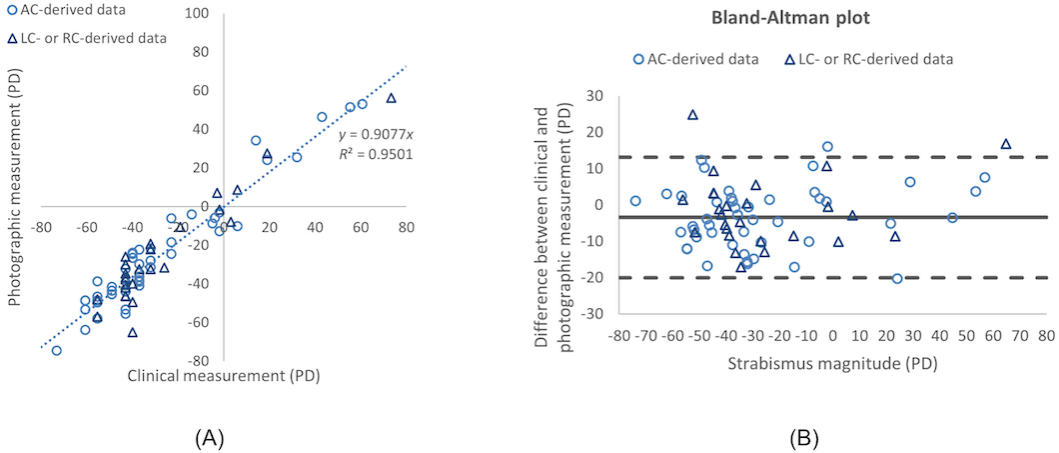
Correlation and agreement between photographic and clinical strabismus measurements. (A) Linear regression analysis comparing strabismus calculation results and physician measurements. (B) Bland-Altman plot for agreement between the strabismus detection system and the physician measurements. In both panels, data points derived from AC images (n=45) are shown as hollow circles, while points derived from unilateral cover (LC or RC) images (n=26) are shown as hollow triangles. AC: alternating cover; LC: left eye cover-uncover; PD: prism diopter; RC: right eye cover-uncover.

In this study, we consider strabismus calculation larger than 104 PD to be an indication of incorrect image processing, since the highest 2 powers of the prism set used by the clinicians were 45 and 40 PD. With the 2 prisms used at the same time (1 prism on each eye), the largest strabismus would be 104 PD according to the lookup table of Cestari and Hunter [39]. None of the measurements in this study were above this threshold. The linear regression analysis and coefficient of determination are shown in Figure 4A, with a determination coefficient (*R*^2^) of 0.95. The slope of the linear regression was close to 1 (0.91). When analyzed separately, the slopes of regression for base out and base in deviations were 0.908 and 0.904, respectively (since they were similar, the data were not separated). As the Bland-Altman plot showed in Figure 4B, the 95% limits of agreement was –20.2 to 14.6 PD.

**Figure 5:**
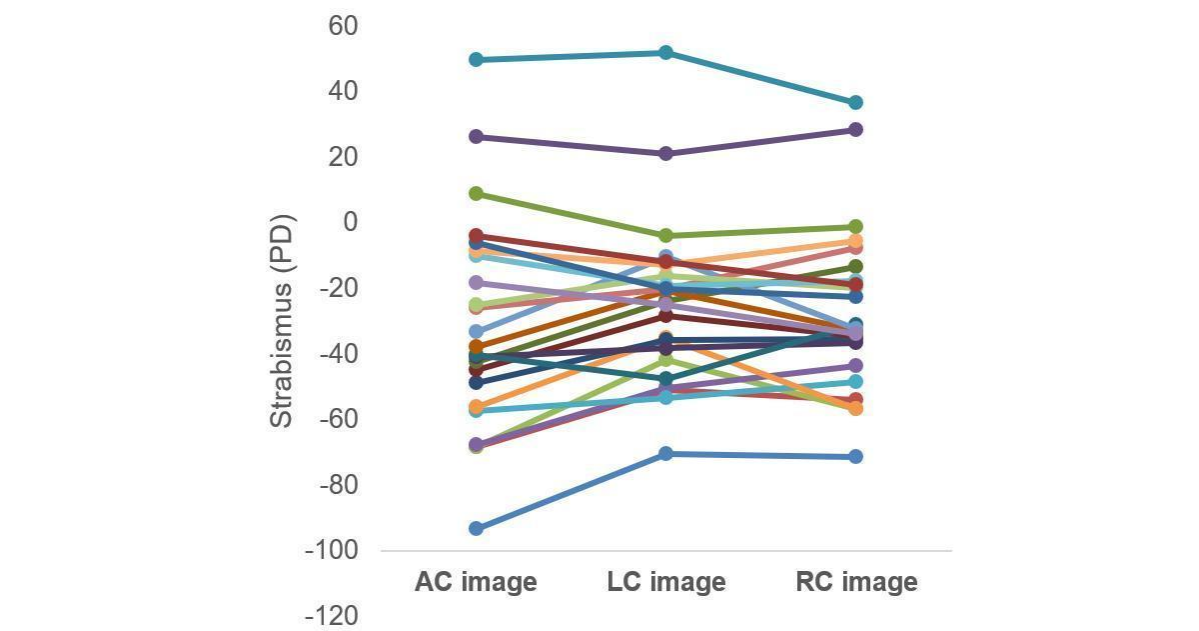
Each line in this graph represents the 3 strabismus calculation results based on AC, LC, and RC images, respectively. Bilateral covering (AC images) revealed larger strabismus than unilateral covering (LC and RC images). AC: alternating cover; LC: left eye cover-uncover; PD: prism diopter; RC: right eye cover-uncover.

Furthermore, image assessment based on different cover methods was compared using repeated measures ANOVA, which revealed a significant difference (*P*=.007; *F*_2_=5.55). The averages of the 3 cover methods were 38.3 (SD 23.7), 30.8 (SD 17.2), and 32.1 (SD 17.8) PD for AC, LC, and RC, respectively. Bilateral cover test (based on AC images) revealed slightly but statistically significantly larger strabismus magnitudes (by about 6 to 8 PD) than unilateral cover test (based on LC and RC images). It is known that bilateral cover can better break binocular fusion. In this analysis, 23 participants who had calculation results for all the AC, LC, and RC images were included (Figure 5). The other participants had at least 1 calculation missing. When analyzed separately, the regression line slopes for AC, LC, and RC were 0.9, 0.92, and 0.97, respectively.

**Figure 6:**
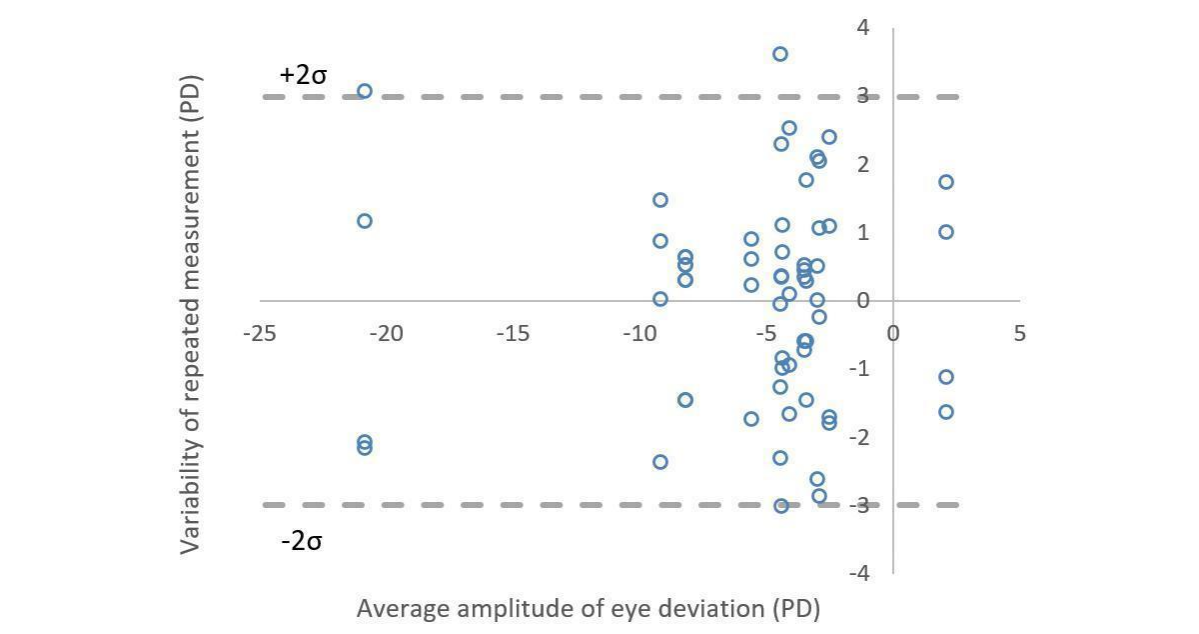
Repeatability of photographic assessment. Each participant (n=15) was photographed 4 times, and the residual errors after subtracting the individual’s average were plotted here. Two dashed lines indicate ±2σ, which represent the 95% CI, ±2.99 PD. PD: prism diopter.

To evaluate the repeatability of Eyeturn Cloud, another set of 15 participants (different than the ones reported earlier), with a mean age of 13.4 (SD 11.5) years (male: n=9 and female: n=6) and including 3 participants with exotropia, 3 with esotropia, and 9 with minimal deviation (<10 PD), were repeatedly photographed 4 times without covering. They were instructed to stare at the flash binocularly when pictures were taken. The variability across trials was calculated for each participant by subtracting the individual average. The SD of the variability was 1.53 PD. Therefore, the 95% CI (2σ) of the measurement was ±2.99 PD (Figure 6). This random noise level can be used as an indicator of the discrimination threshold of the system [40]. In addition, based on the image resolution, the minimum magnitude of phoria detectable (corresponding to 1 pixel for the image resolution of 3024×4032 used in the study) is about 2 PD, which is in a similar range as the measurement variability. Therefore, we rate the smallest amplitude of phoria detectable by the system to be ≈3PD.

## Discussion

### Principal Findings

In this study, we developed and evaluated a web-based platform, Eyeturn Cloud, for strabismus angle calculation. We evaluated the level of agreement between the strabismus angles computed by Eyeturn Cloud based on uploaded pictures of patients’ eyes and standard clinical measurements using prisms. The evaluation showed that the strabismus angles computed by the image-based cloud app had a strong linear correlation with clinical measurements (*R*^2^=0.95; slope=0.91; Figure 4). The measures were obtained from different types of strabismus and for different types of cover conditions.

### Comparison to Prior Work

While the limits of agreement (Figure 4B) may not seem to be small, 2 issues should be noted. First, the repeatability of prism measurement by clinicians may not be very high, depending on deviation amplitude, examiner skill, and patient factor. The Bland-Altman limits of agreement (–20.2 to +14.6 PD) for the cloud app measurements are slightly worse than the interexaminer agreement limits (13 PD reported by de Jongh et al [41], or ±10 PD reported by Hatt et al [42], or ±11.7 PD reported by the Pediatric Eye Disease Investigator Group [43]) for large strabismus angles at near fixation. However, the limits of agreement for the Eyeturn Cloud app with prism testing compare favorably with other photographic strabismus assessment methods. For example, Strabocheck [27] evaluation reported 95% interval limit between –30.0 and 31.0 PD, and Garcia et al [23] reported agreement limits between –15.78 and 24.53 PD.

Second, the moderate error for small phoria of some control participants was due to the incorrect determination of esophoria versus exophoria, which leads to the incorrect sign of results. For instance, an exophoria of 3 PD was interpreted as an esophoria of –7PD. Thus, the disagreement became 10 PD. While it is still within the limits of agreement as mentioned earlier, the incorrect sign can be an error of clinical significance. There were 3 participants with orthophoria (3 PD base out, 6 PD base out, and 3 PD base in) misclassified in data analysis. One of the causes of this problem is that the visual axis is not precisely aligned with the pupil center because of the angle κ. Even when an eye is fixating on the flash, the corneal reflection is off the center nasally. In this study, we applied an offset of 4° angle κ in both eyes of participants with small deviation (<10 PD). As the angle κ of each individual eye was unknown, and because the current Eyeturn Cloud user interface does not have an option to indicate whether the uploaded image was taken with cover or not, or which eye was last uncovered if it was a cover test image, the deviated eye could not be identified simply by the larger offset. However, for relatively large deviations, base out and base in can be reliably determined based on the corneal reflection offset in the deviated eye. The current Eyeturn Cloud does not indicate base out or base in for users when the calculated deviation is smaller than 10 PD, in order to prevent misclassification in actual application. In this paper, the 3 participants with orthotropia were still counted misclassification just for the sake of data analysis. How to determine small esophoria and exophoria correctly is our future work. One possible approach can be through analyzing eye movement during the cover test based on videos. An alternative approach would be implementing a protocol where both eyes are captured monocularly (under unilateral cover conditions) as well as binocular condition (no cover). From the joint analysis of the 3 images, small-angle esotropia and exotropia could be classified with greater accuracy.

The proposed Eyeturn Cloud solution is similar to the Eyeturn smartphone app [19] in terms of the underlying approach of strabismus computation, although the landmarks within the eyes are now detected using AI algorithms. Compared to the 2WIN handheld photoscreening device that can provide quantitative strabismus measures, our approach is different in terms of imaging (visible vs infrared) and processing (iris vs pupil segmentation). Evaluation of the 2WIN portable device in 137 children with manifest strabismus and with dilated pupils showed *R*^2^ values of 0.58 and 0.24 for esotropia and exotropia, respectively, when compared to alternate cover prism testing [21]. The maximum deviation included in that study was <40 PD. In addition, there are some differences between Eyeturn Cloud and previous web-based approaches that compute the strabismus angle from uploaded pictures. Compared to the Strabocheck website [27], which requires 3 pictures (left eye, right eye, and both eyes) and manually marking eye features, our approach can automatically assess strabismus based on a single picture, which can be captured under no cover, unilateral cover for 1 eye, or alternate cover conditions. Compared to the AI-based platform developed by Wu et al [28] for strabismus screening that detects the presence of strabismus from ocular pictures, Eyeturn Cloud provides a quantitative measurement of the eye deviation.

### Strength

The Eyeturn Cloud platform enables the use of general digital devices, like smartphones and cameras, to capture and upload eye images for analysis by a web app in the cloud. This approach not only reduces the need for expensive, specialized equipment but also expands access to screening for a broader population. The platform’s reliance on cloud infrastructure ensures that complex computations are handled remotely, eliminating the processing burden on local devices and allowing for batch processing of large datasets. This capability is particularly advantageous in large-scale screening programs, such as those conducted in schools, communities, or rural areas, where high-volume data processing must be processed efficiently. The ability to upload images offline and process them later when internet access is available is particularly crucial for rural sites.

While in this study, the pictures during unilateral cover or alternate cover testing were captured by a special-purpose cover test smartphone app, it should be noted that this app is not strictly necessary for capturing the appropriate pictures. Users could use any modern native camera apps (eg, live photo feature of iPhone camera app) or record a high-resolution (4K) video, and then, select the required frame when the eye is first visible after removal of the cover (see demonstration in Multimedia Appendix 1). In the future, the Eyeturn Cloud app could be updated to accept high-resolution videos and automatically select the appropriate frame based on the visibility of both eyes to compute strabismus angles. Furthermore, for constant strabismus assessment, cover is not needed. Thus, pictures taken with regular cameras can be processed by Eyeturn Cloud to yield valid results.

One potential advantage of cloud AI platforms is that they can facilitate the maintenance of digital archives of patient data, allowing ophthalmologists to monitor the progression of strabismus over time and adjust their treatment plans accordingly. Such a data-driven approach has the potential to enhance diagnostic accuracy and promote more personalized care, ultimately improving patient outcomes through timely interventions and continuous analysis. In addition, these AI platforms may support telemedicine-based screening and follow-up care [14,15], which is especially beneficial in areas lacking access to specialized ophthalmic equipment. This reduces the need for frequent patient visits and facilitates consistent remote monitoring [44]. Regarding screening, the Eyeturn smartphone app was previously used for strabismus screening in school children in a pilot study [45]. As the underlying computation is conceptually similar, it is reasonable to expect that Eyeturn Cloud could also be potentially useful in such screening settings. However, the utility of Eyeturn Cloud in screening and telehealth settings is yet to be determined and is future work.

### Limitations

There are still some unresolved issues with the current app. The cloud app was able to generate valid strabismus results for 71 patients, while the remaining 8 patients could not yield results. Among these, the results were invalid due to the absence of corneal light reflex points or incorrect iris segmentation by SAM. The issue of missing corneal light reflex points could potentially be resolved by selecting an appropriate environment for photography or by attempting multiple captures. On the other hand, eyes with glasses were excluded in this study, as the flash could interfere with the glasses causing spurious reflections, if caution was not taken during picture capturing. This could be solved in the future by more intelligent algorithms than simple thresholding methods.

In theory, our method could reliably measure ocular misalignment above 3 PD, but determining the directionality of misalignment is a challenge for photoscreening based on a single image. To mitigate sign instability near 0, the app withholds directionality when the estimated magnitude is <10 PD. Despite lack of directionality, strabismus magnitude measurement can still be useful information for vision screening.

There are also some limitations of this study. All of the pictures included in this study were all taken with our cover test app by clinicians in controlled settings rather than by lay people. This environment likely ensured optimal lighting, correct camera distance, and maximal patient cooperation. The usability, performance, and reliability of the Eyeturn Cloud platform when used by laypersons, such as parents at home for a telehealth consultation or by school nurses in a community screening program, remain unknown. In addition, pictures captured using other acquisition methods, such as native camera apps on other smartphones, have not been evaluated. Another limitation was that the number of base in deviation was higher than base out in our participants, due to the biased prevalence in the population [46], and future studies need to consider balancing the sample for more thorough analysis of app’s performance for base out deviations. The study only evaluated images captured along primary gaze direction, and differentiating incomitant and concomitant strabismus and measuring the deviation patterns are future work. However, the findings of this study could guide future studies, where its utility for telehealth and screening applications could be fully evaluated.

### Conclusions

The main novelty lies in the approach where we developed the cloud-AI app for strabismus angle computation based on a single picture, which can be acquired from different sources. While AI tools exist for strabismus screening from a single image [28], our app focuses on quantitative measurement of angular deviation. Our proposed solution was intended to address scenarios where a cover test is needed as well as to offer a possibility to assess photos captured with regular cameras without covering. Cover-testing mode is important for measuring patients with nonmanifest strabismus. The cloud-AI platform treats all image sources in the same way. Overall, the Eyeturn Cloud offers a convenient and accessible way of strabismus assessment. This study showed that quantitative assessment of strabismus with Eyeturn Cloud was repeatable and demonstrated a strong correlation with clinical measurements, although further work is required to improve its precision and address misclassifications of small-angle deviations. While the platform’s architecture suggests potential for future application in vision screening and telehealth, its efficacy and reliability in these real-world settings, particularly when used by nonexperts with varied hardware, require rigorous future investigation before any such use can be recommended.

## Appendix

Multimedia Appendix 1 Demonstration video.

Multimedia Appendix 2 MAIC-10 brief quality checklist.

## Abbreviations

AC: alternating cover
AI: artificial intelligence
AWS: Amazon Web Services
BCVA: best‑corrected visual acuity
LC: left eye cover-uncover
PD: prism diopter
RC: right eye cover-uncover
SAM: Segment Anything Model
YOLO: You Look Only Once
