# Supplementary material for: Quantitative Assessment of Strabismus Using Cloud AI Computing: Validation Study
Source: JMIR Form Res. 2025 Nov 4;9:e79280. doi: 10.2196/79280 (PMC12627973; doi:10.2196/79280)
Supplement: Multimedia Appendix 2 [file formative_v9i1e79280_app2.pdf]

Item No.	Item name	Item description	Score	
1	Clinical need	The study is clearly put into context by describing the target clinical problem and any previous approaches in the literature.	1	Introduction / Discussion
2	Study design	The type of study (observational/interventional, single/multicentre) and inclusion/exclusion criteria are described explicitly, and a sample size estimate is given.	1	Methods (Evaluation subsection)
3	Safety and privacy	ELSI (Ethical, Legal, Social Implications), specifically including ethics committee approval and data de-identification issues, are discussed.	1	Methods (Evaluation subsection)
4	Data curation	Data extraction, cleaning and transformation methods, including image pre-processing steps, are clearly	1	Methods
5	Data annotation	The ground truth reference is defined and the annotation process, including measures of inter/intra-observer variability, is described.	0	
6	Data partitioning	Methods and criteria for data set splitting into train-tune-test-validation sets are indicated.	1	Methods (Cover test app subsection)
7	AI model	The AI model building methodology is sufficiently detailed by including used technologies (software and hardware), training-tuning-testing methods, performance metrics, and resulting AI model architecture.	1	Methods (Pipeline of strabismus calculation, Cover Test app subsections)
8	Robustness	The generalisability of the AI model in real-world conditions, or lack thereof, is explicitly discussed.	1	Throughout the article
9	Explainability	The transparency and interpretability of the model (including the use of uncertainty or confidence metrics), or lack thereof, is explicitly discussed.	0	
10	Transparency	Any possibility of access to original data sets and source code is clearly stated. Financing and conflicts of interest are detailed.	1	Funding statement, Competing interests, Data sharing

		overall score	8	

Citation: Cerdá-Alberich L, Solana J, Mallol P, Ribas G, García-Junco M, Alberich-Bayarri A, Marti-Bonmati L. MAIC-10 brief quality checklist for publications using artificial intelligence and medical images. Insights Imaging. 2023 Jan 16;14(1):11. doi: 10.1186/s13244-022-01355-9. PMID: 36645542; PMCID: PMC9842808.
